# Genome-wide identification and analysis of A-to-I RNA editing events in the malignantly transformed cell lines from bronchial epithelial cell line induced by α-particles radiation

**DOI:** 10.1371/journal.pone.0213047

**Published:** 2019-06-03

**Authors:** Qiaowei Liu, Hao Li, Lukuan You, Tao Li, Lingling Li, Pingkun Zhou, Xiaochen Bo, Hebing Chen, Xiaohua Chen, Yi Hu

**Affiliations:** 1 Medical School of Chinese PLA, Beijing, P.R. China; 2 Department of Medical Oncology, Chinese PLA General Hospital, Beijing, P.R. China; 3 Beijing Institute of Radiation Medicine, Beijing, P.R. China; CNRS UMR7622 & University Paris 6 Pierre-et-Marie-Curie, FRANCE

## Abstract

Adenosine (A) to inosine (I) RNA editing is the most prevalent RNA editing mechanism in humans and plays critical roles in tumorigenesis. However, the effects of radiation on RNA editing were poorly understood, and a deeper understanding of the radiation-induced cancer is imperative. Here, we analyzed BEP2D (a human bronchial epithelial cell line) and radiation-induced malignantly transformed cell lines with next generation sequencing. By performing an integrated analysis of A-to-I RNA editing, we found that single-nucleotide variants (SNVs) might induce the downregulation of ADAR2 enzymes, and further caused the abnormal occurrence of RNA editing in malignantly transformed cell lines. These editing events were significantly enriched in differentially expressed genes between normal cell line and malignantly transformed cell lines. In addition, oncogenes *CTNNB1* and *FN1* were highly edited and significantly overexpressed in malignantly transformed cell lines, thus may be responsible for the lung cancer progression. Our work provides a systematic analysis of RNA editing from cell lines derived from human bronchial epithelial cells with high-throughput RNA sequencing and DNA sequencing. Moreover, these results provide further evidence for RNA editing as an important tumorigenesis mechanism.

## Introduction

Lung cancer is the most frequent cancer and the leading cause of cancer death among males[1], and radon exposure is the second most common cause of lung cancer after smoking [2–7]. However, the molecular mechanisms of radon-induced lung cancer remain unclear.

RNA editing is a post-transcriptional modification process, the deamination of adenosines (A) to inosines (I) is the prominent RNA editing event in humans, where ADAR enzymes convert A to I and destabilize double-stranded without affecting the DNA sequence identity [8]. Intriguingly, RNA editing plays an important role in tumorigenesis, such as recoding RNA editing of AZIN1 predisposes to hepatocellular carcinoma[9], RNA editing in RHOQ promotes invasion potential in colorectal cancer [10], and *GABRA3* editing suppresses breast cancer metastasis [11]. Recent study has shown that, in non-small cell lung cancer samples, as a result of ADAR gene amplification, the RNA editing of DNA repair enzyme NEIL1 (K242R) was increased recoding [12]. However, there are limited studies to date in further exploring the characteristics of RNA editing in lung cancer. What’s more, there are few reports about the effect of radiation on RNA editing.

Here, we investigated A-to-I RNA editing in human bronchial epithelial cell line (BEP2D) and malignantly transformed cell lines (BERP35T1 and BERP35T4), which were important models to characterize the radiation-mediated carcinogenesis of lung [13, 14]. By performing high-throughput RNA sequencing, we identified A-to-I editing sites with three robust bioinformatics methods. We then systemically compared editing events in normal line and malignantly transformed cell lines. Further, by performing genome-wide DNA sequencing, we revealed that the genomic variants in *ADAR2* gene were correlated with the abnormal editing events in malignantly transformed cell lines. Finally, we reported two potential edited genes, *CTNNB1* and *FN1*, in malignantly transformed cell lines.

## Results

### Identification of A-to-I RNA editing

**Fig 1:**
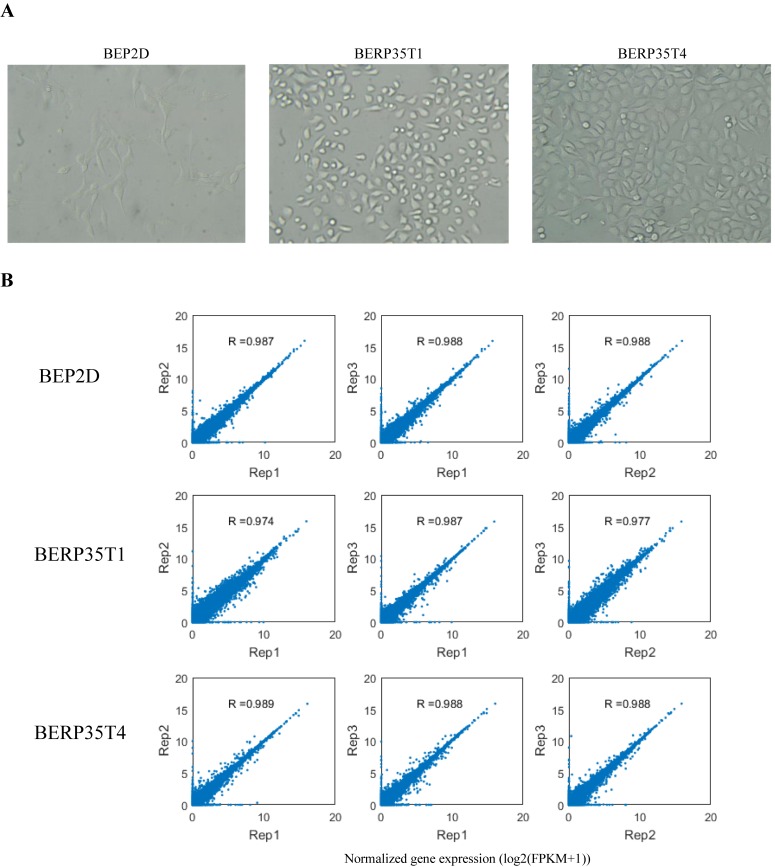
Cell culture and RNA sequencing. **A.** Photomicrographs of BEP2D (Left), BERP35T1 (Middle) and BERP35T4 (Right) showing the cellular atypia of the malignant transformed cell lines (100×). **B.** Scatter plots showing the consistency of normalized gene expression between biological replicates for each cell line.

The prevalence and importance of A-to-I RNA editing have been illuminated in recent years largely owing to the rapid adoption of high-throughput sequencing technologies [15, 16]. To analyze A-to-I RNA editing in BEP2D cell line and malignantly transformed cell lines, we performed high-throughput RNA sequencing (RNA-Seq) on BEP2D cell line and transformed BEP2D cell lines, which were irradiated with 1.5Gy dose of α-particles emitted by ^238^PuO_2_. Two transformed cell lines, BERP35T1 and BERP35T4, were investigated (Fig 1A). Three biological replicates were sequenced and analyzed for each cell line. We then calculated gene expression level with Cufflinks program [17], for each cell line, biological replicates of RNA-seq revealed highly reproducible gene expression (Fig 1B). Thus, our sequencing data were of high quality for RNA editing identification and gene expression analysis.

Recent studies have reported that the most challenging part of identifying RNA editing is the discrimination of RNA editing sites from genome-encoded single-nucleotide polymorphisms (SNPs) and technical artifacts caused by sequencing or read-mapping errors [18–20]. To accurately identify RNA editing sites, we performed three widely-used methods including GIREMI [21], RNAEditor [22] and Separate method from Jin Billy Li [18] (See Methods). The GIREMI method combines statistical inference of mutual information (MI) between pairs of single-nucleotide variants (SNVs) in RNA-seq reads with machine learning to predict RNA editing sites. RNAEditor calls RNA editing by detecting ‘editing islands’. Separate method from Jin Billy Li identifies RNA editing sites by strict filtering processes. For each sample, we only used RNA editing sites that can be detected in all three methods. For each cell line, we combined RNA editing sites from three biological replicates. As the most prevalent editing type in humans is adenosine-to-inosine (A-to-I) editing and most noncanonical editing are false positives [23], we only analyzed A-to-I RNA editing in this study. Final, 5659, 3820 and 2446 A-to-I RNA editing sites were identified in BEP2D cell line and transformed cell lines BERP35T1 and BERP35T4, respectively (Table 1 and S1 Table).

**Table 1:** Summary of A-to-I RNA editing.

Sample	GIREMI	JinBilly	Editor	All methods	RNA editing
BEP2D REP1	3721	13139	20585	2822	5659
BEP2D REP2	3712	13776	20322	2892	
BEP2D REP3	3982	15356	22106	3078	
BERP35T1 REP1	1929	7693	10877	1429	3820
BERP35T1 REP2	3355	13258	18762	2496	
BERP35T1 REP3	1990	9081	12728	1500	
BERP35T4 REP1	1746	7587	10895	1302	2446
BERP35T4 REP2	1537	7088	9948	1122	
BERP35T4 REP3	1603	6597	9317	1202	

### A-to-I RNA editing and associated genes in BEP2D cell line and malignantly transformed cell lines

**Fig 2:**
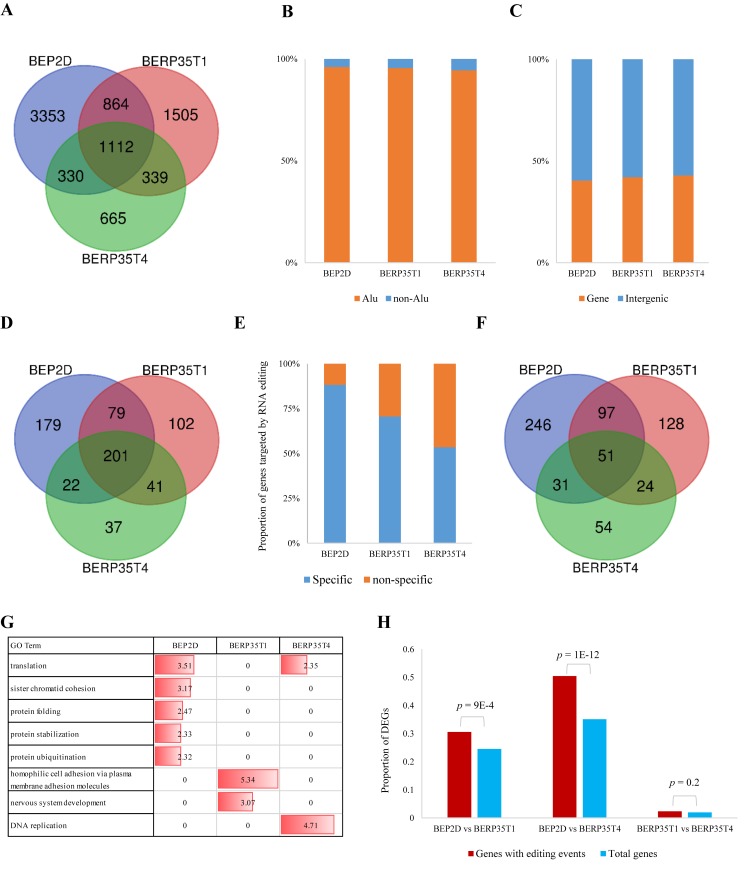
Identification and characterization of A-to-I RNA editing. **A.** Venn plot showing the overlaps of RNA editing sites in BEP2D, BERP35T1 and BERP35T4 cell lines. **B, C.** Bar plots showing the proportion of A-to-I RNA editing in Alu/non-Alu regions (B) and Genebody/Intergenic regions (C). **D.** Venn plot showing the overlaps of edited genes in BEP2D, BERP35T1 and BERP35T4 cell lines. **E.** Bar plot showing the proportion of tissue-specific editing genes in each cell line. **F.** Venn plot showing the overlaps of edited genes contained cell line-specific editing sites in BEP2D, BERP35T1 and BERP35T4 cell lines. **G.** GO enrichment for editing genes in each cell line, value was negative log10 of *p*-value. **H.** We compared the proportion of DEGs in total genes (blue bar) and DEGs in editing genes (red bar), *p*-value was calculated by hypergeometric test.

We next investigated the difference of A-to-I RNA editing between BEP2D cell line and malignantly transformed cell lines. First, 3,683~4,217 editing events in BEP2D cell line were not detected in malignantly transformed cell lines, and 1,004~1,844 new editing events occurred from normal BEP2D cell to malignantly transformed cells, indicating dramatic changes of RNA editing when BEP2D cell line was irradiated (Fig 2A), gene expression changes induced by radiation may cause this change. Generally, A-to-I editing is pervasive in Alu repeats because of the double-stranded RNA structures formed by inverted Alu repeats in many genes [24, 25]. We found that although RNA editing was quite different in BEP2D and malignantly transformed cell lines, editing sites were still conserved in Alu repeats (Fig 2B) and ~60% of RNA editing events occurred in intergenic regions (Fig 2C). Thus, the proportions of A-to-I RNA editing located in Alu and intergenic were not change after radiation.

Next, we examined genes targeted by A-to-I RNA editing sites (edited genes for short). In general, 484, 426 and 305 genes were edited in BEP2D, BERP35T1 and BERP35T4 cell lines (Fig 2D and S2 Table). We found that, in BEP2D cell line, 88% of edited genes contained BEP2D-specific editing sites, but in BERP35T1 and BERP35T4 cell lines, only 70% and 53% of edited genes contained cell-specific editing sites (Fig 2E and Fig 2F). This result suggested that the editing rate of genes decreased when cell was irradiated and malignantly transformed. In addition, we performed gene ontology (GO) analysis to reveal the biological function of editing genes. We found that editing genes were enriched in different biological processes. For BEP2D cell line, editing genes were enriched in protein processes and translation process, for BERP3T1 cell line, nervous system development and hemophilic cell adhesion process were highlighted and editing genes were enriched in DNA replication in BERP35T4 cell line (Fig 2G).

To examine whether RNA editing affects transcription activity, we identified differentially expressed genes (DEGs) by performing Cuffdiff program [17] (S3 Table). We found that DEGs between normal BEP2D cell line and malignantly transformed cell lines were significantly enriched in genes with RNA editing events (*p* value < 1E-3, hypergeometric test, Fig 2H). This observation revealed that there was a significant correlation between RNA editing events and gene dysregulation.

### *ADAR2* down-regulation by genome SNVs

**Fig 3:**
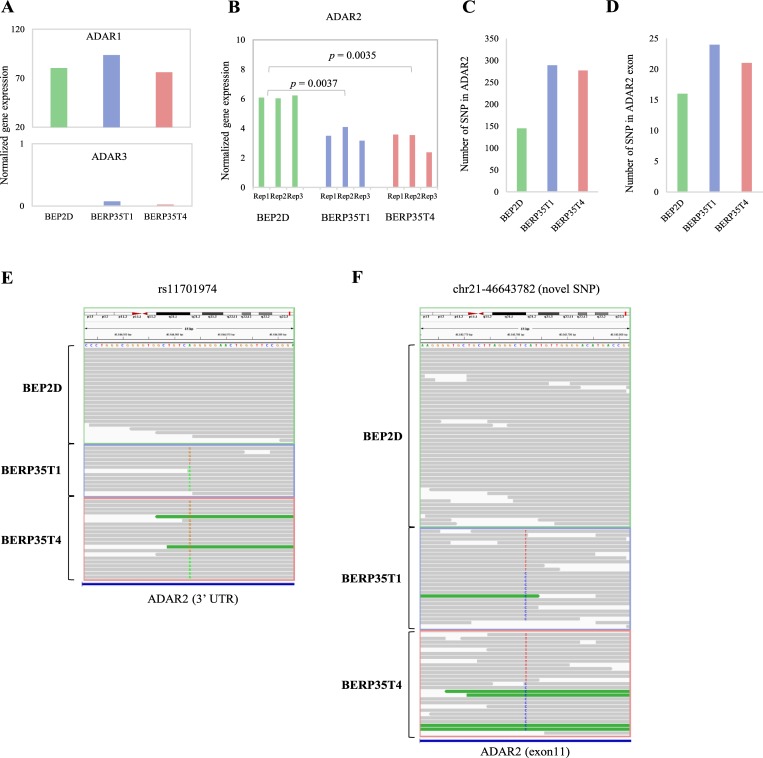
Down-regulation of ADAR2 induce RNA editing. **A, B.** Bar plots showing the normalized gene expression of *ADAR1*, *ADAR2* and *ADAR3* in each cell line. **C, D.** Bar plots showing the number of genomic variants in *ADAR2* (C) and exons of *ADAR2* (D). **E, F.** IGV plots showing the sequencing reads information of mutation rs11701974 and novel mutation chr21:46643782.

We next investigated the mechanism responsible for the differences observed in RNA editing between normal BEP2D cell line and malignantly transformed cell lines. In human, A-to-I editing is performed by the ADAR family, which contains 3 genes: *ADAR1*, *ADAR2* and *ADAR3* [26–28]. We thus examined the transcript levels of *ADAR* genes. The expression level of *ADAR1* in BEP2D cell line was comparable to that in BERP35T1 and BERP35T4 cell lines and *ADAR3* was silence in both BEP2D cell line and malignantly transformed cell lines (Fig 3A). However, *ADAR2* expression level significantly reduced in BERP35T1 and BERP35T4 cell lines (Fig 3B). Previous studies have confirmed that *ADAR2* is lowly expressed in cancer e.g. glioblastoma [29], gastric cancer[30]. Thus, the tumor progression of BEP2D seems to mainly be induced by *ADAR2* downregulation.

We further explored the possible mechanism of *ADAR2* downregulation in malignantly transformed cell linea. Radiation induced DNA alterations change gene expression and further increase cancer risk [31, 32]. We next performed DNA sequencing (DNA-seq) on BEP2D cell line and malignantly transformed cell lines. We identified single-nucleotide variants (SNVs) using GATK pipeline [33] (See Methods). Surprisingly, nearly 2-fold SNVs in *ADAR2* gene were detected in malignantly transformed cell lines compared to normal BEP2D (Fig 3C and S4 Table) and more SNVs in *ADAR2* exon were observed (Fig 3D). For example, known mutation rs11701974, a genetic variant of HLA-DQB1 associated with human longevity [34], was detected in 3’ UTR of *ADAR2* and specific in BERP35T1 cell line and BERP35T4 cell line (Fig 3E). Moreover, we identified 32 and 24 novel mutations in BERP35T1 cell line and BERP35T4 cell line, respectively (S4 Table). For example, chr21:46643782 was altered in malignantly transformed cell lines (Fig 3F). These results indicate that radiation leads to SNVs, and this may further relate to the dysregulation of ADAR2, which needs to be examined in the future.

### Oncogene *CTNNB1* and *FN1* are highly edited and significantly overexpressed in malignantly transformed cell line

**Fig 4:**
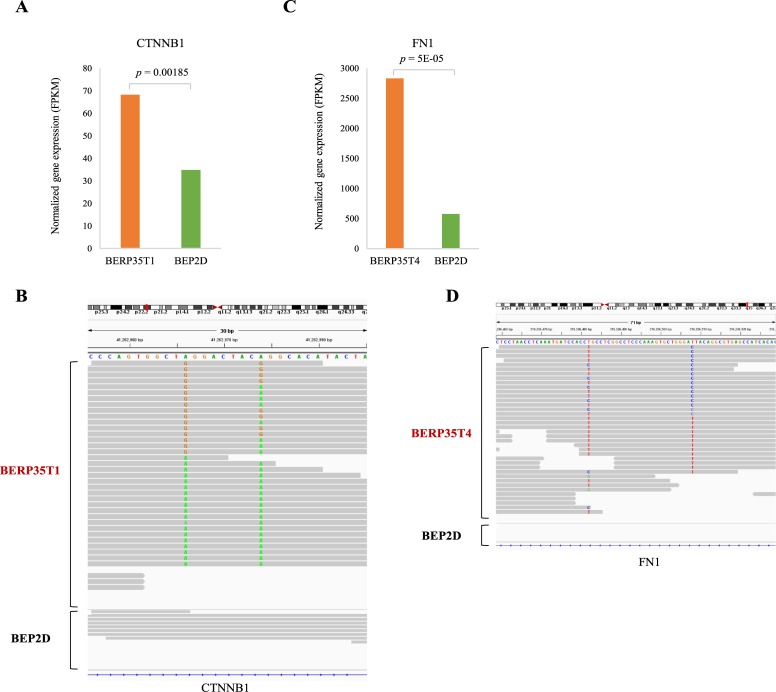
Oncogene CTNNB1 and FN1 are highly edited and significantly overexpressed in malignantly transformed cell line. **A, C.** Bar plots showing the normalized gene expression of oncogenes *CTNNB1* and *FN1* in BEP2D cell line and BERP35T1 and BERP35T4 cell lines, respectively. **B, D.** IGV plots showing the editing events in oncogenes *CTNNB1* and *FN1*.

To gain insights into the biological relevance of RNA editing in malignantly transformed cell lines, we investigated 285 oncogenes from previous studies [35] (S5 Table). We found that oncogenes *CTNNB1*, *PABPC1* and *VHL* were edited in BERP35T1 cell line. Notably, the expression level of *CTNNB1* in BERP35T1 cell line was significantly higher than that in BEP2D cell line (*p*-value = 0.00185, Fig 4A). Previous studies reported that activating mutations in *CTNNB1* have oncogenic activity resulting in tumor development and somatic mutations are found in various tumor types [36–39]. We found two A-to-I editing events (chr3:41262966 and chr3:41262974) occurred in BERP35T1 cell line and *CTNNB1* was overexpression in BERP35T1 cell line (Fig 4B). Similarly, we found three oncogenes *FN1*, *METTL14* and *VHL* were edited in BERP35T4 cell line. Notably, the expression level of *FN1* in BERP35T4 was significantly higher than that in BEP2D cell line (*p*-value = 5E-5, Fig 4C). Previous studies have reported that transcriptional activation of *FN1* and gene fusions of *FN1* promote the malignant behavior of multiple cancers [40–42]. A strong A-to-I editing event (chr2:216236508) and a weak A-to-I editing event (chr2:216236482) were observed in BERP35T4 and *FN1* was overexpression in BERP35T4 cell line (Fig 4D). These results suggest that RNA editing is associated with oncogene overexpression and may further induce cancer progression.

## Discussion

Radon is a recognized cause of lung cancer, however, the cellular and molecular mechanisms of radon-induced lung cancer remains unknown. To facilitate the study of this question, in our previous work, we established a model system of α-particle transformed human cell lines [43]. Then, we found a number of alterations in these cell models, including cytogenetics [44, 45], gene expression [46], DNA repair [14], and genomic instability [47]. However, a genome-wide systematic analysis of this model based on next generation of sequencing is absent. In this work, we performed high-throughput RNA sequencing and genome-wide DNA sequencing to this model and discovered a new mechanism probably for tumorigenesis.

While the oncogenic effect of DNA damage induced by radiation has been illuminated, the effects of radiation on RNA editing are unclear in tumorigenesis. In this work, we provided genome-wide identification and analysis of A-to-I RNA editing events in the malignantly transformed cell lines induced by α-particles radiation, the results show that RNA editing sites change greatly and the total amount decreased after radiation.

RNA editing plays an important role in post-translational modification which can affect mRNA’s structure and stability [46, 48], but little is known about how RNA editing operates in cancer [49]. Our work supposes that, in these cell models, SNVs may cause the downregulation of ADAR2 enzymes, and further caused the abnormal occurrence of RNA editing in malignantly transformed cell lines. This hypothesis needs to be confirmed in the future. Then, the abnormal occurrence of RNA editing led to abnormal expression of oncogenes, such as, *CTNNB1* and *FN1*, thus may be responsible for the lung cancer progression. These results demonstrate further evidence for RNA editing as an important tumorigenesis mechanism, and RNA editing sites might represent a new class of therapeutic targets.

## Materials and methods

### Cell culture

The BEP2D cell line is a human papilloma-virus (HPV18)-immortalized human bronchial epithelial cell line and was established by Dr Curtis C. Harris (National Cancer Institute, MD, USA) [50]. These cells are anchorage dependent and non-tumorigenic in late passages. We got the authorization for research use only from Dr Curtis C. Harris and the Passage 20 of the BEP2D cell line was kindly provided by Tom K Hei (Center for Radiological Research, College of Physician and Surgeons, Columbia University, New York, USA) in the summer of 1993. The authentication of BEP2D cell line was tested by using short tandem repeats (STRs) analysis in June 2018. Although the information of BEP2D cell line was not found in DSMZ and ATCC, our STRs results did match BBM cell line (ATCC Cell No. CRL-9482), BZR cell line (ATCC Cell No. CRL-9483) and BEAS-2B cell line (ATCC Cell No. CRL-9609), which are three tranformants derived from human bronchial epithelial cell (S1 File). The BERP35T1 and BERP35T4 malignant transformant cell lines were derived from BEP2D cell line irradiated with 1.5 Gy of α-particle emitted from ^238^Pu source and were described in detail in a previous paper[14]. The cells were cultured in serum‑free LHC‑8 medium (Gibco, USA) at 37˚C under a 95% air/5% CO_2_ atmosphere.

### RNA sequencing

Total RNAs were extracted from cells with RNAiso Reagent (TaKaRa, Dalian, China) following the manufacturer’s instruction. RNA degradation and contamination were monitored on 1% agarose gels. RNA purity was checked using the NanoPhotometer spectrophotometer (IMPLEN, CA, USA). RNA concentration was measured using Qubit RNA Assay Kit in Qubit 2.0 Flurometer (Life Technologies, CA, USA). RNA integrity was assessed using the RNA Nano 6000 Assay Kit of the Bioanalyzer 2100 system (Agilent Technologies, CA, USA). A total amount of 1 μg RNA per sample was used as input material for the RNA sample preparations. Sequencing libraries were generated using NEBNext Ultra^TM^ RNA Library Prep Kit for Illumina (NEB, USA) following manufacturer’s recommendations and index codes were added to attribute sequences to each sample. Briefly, mRNA was purified from total RNA using poly-T oligo-attached magnetic beads. Fragmentation was carried out using divalent cations under elevated temperature in NEBNext First Strand Synthesis Reaction Buffer (5X). First strand cDNA was synthesized using random hexamer primer and M-MuLV Reverse Transcriptase (RNase H-). Second strand cDNA synthesis was subsequently performed using DNA Polymerase I and RNase H. Remaining overhangs were converted into blunt ends via exonuclease/polymerase activities. After adenylation of 3’ ends of DNA fragments, NEBNext Adaptor with hairpin loop structure were ligated to prepare for hybridization. In order to select cDNA fragments of preferentially 150~200 bp in length, the library fragments were purified with AMPure XP system (Beckman Coulter, Beverly, USA). Then 3 μl USER Enzyme (NEB, USA) was used with size-selected, adaptor-ligated cDNA at 37°C for 15 min followed by 5 min at 95°C before PCR. Then PCR was performed with Phusion High-Fidelity DNA polymerase, Universal PCR primers and Index (X) Primer. At last, PCR products were purified (AMPure XP system) and library quality was assessed on the Agilent Bioanalyzer 2100 system.

The clustering of the index-coded samples was performed on a cBot Cluster Generation System using TruSeq PE Cluster Kit v3-cBot-HS (Illumia) according to the manufacturer’s instructions. After cluster generation, the library preparations were sequenced on an Illumina Hiseq platform and 150 bp paired-end reads were generated.

### DNA sequencing

Total DNAs were extracted from cells with DNAiso Reagent (TaKaRa, Dalian, China) following the manufacturer’s instruction. The quality of isolated genomic DNA was verified by using these two methods in combination: (1) DNA degradation and contamination were monitored on 1% agarose gels. (2) DNA concentration was measured by Qubit DNA Assay Kit in Qubit 2.0 Flurometer(Life Technologies, CA, USA). A total amount of 1μg DNA per sample was used as input material for the DNA library preparations. Sequencing library was generated using Truseq Nano DNA HT Sample Prep Kit (Illumina, USA) following manufacturer’s recommendations and index codes were added to each sample. Briefly, genomic DNA sample was fragmented by sonication to a size of 350 bp. Then DNA fragments were endpolished, A-tailed, and ligated with the full-length adapter for Illumina sequencing, followed by further PCR amplification. After PCR products were purified (AMPure XP system), libraries were analyzed for size distribution by Agilent 2100 Bioanalyzer and quantified by real-time PCR (3nM).

The clustering of the index-coded samples was performed on a cBot Cluster Generation System using Hiseq PE Cluster Kit (Illumina) according to the manufacturer’s instructions. After cluster generation, the DNA libraries were sequenced on Illumina Hiseq platform and 150 bp paired-end reads were generated.

### RNA editing identification

We adopted three previously published methods to accurately identify A-to-I RNA editing sites.

For Jinbilly’s method [18], we used the Burrows-Wheeler algorithm (BWA)[51] to align RNA-seq reads to a combination of the reference genome (hg19) and exonic sequences surrounding known splice junctions from available gene models. We chose the length of the splice junction regions to be slightly shorter than the RNA-seq reads to prevent redundant hits. Picard (http://picard.sourceforge.net/) was then used to remove identical reads (PCR duplicates) that mapped to the same location. GATK tools were used to perform local realignment around insertion and/or deletion polymorphisms and to recalibrate base quality scores. Variant calling was performed using GATK UnifiedGenotyper tool with options stand_call_conf of 0 and stand_emit_conf of 0. Further filtering were performed as described [52].

For GIREMI method [21], RNA-seq mapping and preprocessing was same as Jinbilly’s method. For each mismatch position, a total read coverage of ≧5 was required and the variant allele was required to be present in at least three reads. We then the following types of mismatches: those located in simple repeats regions or homopolymer runs of ≧5 nt, those associated with reads substantially biased toward one strand, those with extreme variant allele frequencies (>95% or <10%) and those located within 4 nt of a known spliced junction. Finally, we perform GIREMI tool to call RNA editing.

For RNAEditor [22], fastq format files from RNA-seq data were directly used as input for RNAEditor tools to call RNA editing sites.

### SNV identification

We used the Bowtie2 [53] to align DNA-seq reads to reference genome hg19, Picard (http://picard.sourceforge.net/) was then used to remove identical reads (PCR duplicates) that mapped to the same location. GATK tools were used to perform local realignment around insertion and/or deletion polymorphisms and to recalibrate base quality scores. Variant calling was performed using GATK UnifiedGenotyper tool.

### Statistical analysis

Gene expression was calculated using Cufflinks program default parameters. Differentially expressed genes (DEGs) were identified by Cuffdiff program, three biological replicates for each cell line were combined as the input of Cuffdiff and a *p*-value was reported to show the significance of DEGs.

RNA editing targeted genes were assigned with ‘bedops’ program. GO analysis were performed by using DAVID [54].

### Accession numbers

The sequencing data have been deposited with the Gene Expression Omnibus under the accession ID GSE126723.

## Supporting information

S1 Table RNA editing sites in each cell line. (XLSX)

S2 Table Genes with RNA editing sites (sheet 1) and genes with tissue-specific RNA editing sites (sheet 2). (XLSX)

S3 Table Differentially expressed genes. (XLSX)

S4 Table SNVs in gene ADAR2 for each cell line. (XLSX)

S5 Table Oncogenes used in this study. (XLSX)

S1 File Report of Cell Line Authentication. (PDF)
