# Supplementary material for: Genome-wide identification and analysis of A-to-I RNA editing events in the malignantly transformed cell lines from bronchial epithelial cell line induced by α-particles radiation
Source: PLoS One. 2019 Jun 3;14(6):e0213047. doi: 10.1371/journal.pone.0213047 (PMC6546236; doi:10.1371/journal.pone.0213047)
Supplement: S1 File — (PDF) [file pone.0213047.s006.pdf]

Report of Cell Line Authentication

Service Code： LWXB18023

Report Date： 2018.6.7

BGI

Project Information			
Project No.	LWXB18023	Project Name	BEP2D cell line authentication
Customer Name	Qiaowei Liu	Customer Unit	Beijing Institute of Radiation Medicine
Customer Contact	dr.jackielau@hotmail.com	PM	ShuangyuWang
Sale name	YamingChen		
**Overview of Services**			
Testing Date	2018.6.4	Report Date	2018.6.7
Sample Number	1		
Actual receivable sum	RMB800		
**Note**			

Catalog

1. Experiment Objectives.....3

2. Experimental procedure and method.....4

2.1 Experiment Reagents.....4

2.2 Experiment Apparatus.....4

2.3 Experimental Procedure.....4

2.4 Detection.....4

3. Results and Data.....5

3.1 BEP2D-Lqw STR Data.....5

3.2 BEP2D-Lqw STR Loci Data Comparison.....6

3.3 BEP2D-Lqw STR Profiles.....6

4. Reference.....6

1. Experiment Objectives

Cell information identification

2. Experimental procedure and method

2.1 Experiment Reagents

HUMDNA TYPING(Yanhuang)

2.2 Experiment Apparatus

GeneAmp® PCR system9700， ABI3730XL

2.3 Experimental Procedure

PCR amplification system:

ddH2O	2	µl
STR21 2X Master Mix	5	µl
STR21 4X Primer Pair Mix	2	µl
DNA	1	µl
Total	10	µl

PCR amplification reaction procedures: Applied Biosystems 9700 PCR System

91℃	1 min	
95℃	10 sec	×28Cycle
58℃	1min	×28Cycle
70℃	20 sec	×28Cycle
60℃	30 min	
4℃	∞	

2.4 Detection

Amplified products were separated using an Applied Biosystems® 3730XL Genetic Analyzer. Experimental Process： Amplified products— Add Internal Lane Standard—template degeneration—Detection(Pre-electrophoresis: 1.2kV 5min, Electrophoresis :7.5kV 2h)—Analysis.

STR-500	0.5µl
PCR products	1µl
HIDI	8.5µL
Total	10µL

3. Results and Data

3.1 BEP2D-Lqw STR Data

STR Loci	Results	
Yindel	2	
AMEL	X	Y
D3S1358	15	17
D13S317	13	
D7S820	10	13
D16S539	12	
SE33	20.2	28.2
D10S1248	14	15
D5S818	12	13
D21S11	28	30
TPOX	6	11
D1S1656	14	
D6S1043	12	18
DXS6795	11	
D19S433	13.2	15.2
D22S1045	14	16
D8S1179	13	15
Penta E	5	8
DYS391	10	
D2S441	11	11.3
D12S391	17	18
D2S1338	22	23
vWA	17	18
Penta D	2.2	13
TH01	7	9.3
D18S51	18	19
CSF1PO	9	12
FGA	20	24

3.2 BEP2D-Lqw STR Loci Data Comparison

Search result in DSMZ database:

EV	Cell No.	Cell name	Locus names								
			D5S818	D13S317	D7S820	D16S539	VWA	TH01	AM	TPOX	CSF1PO
	Query (Your Cell)		12,13	13,13	10,13	12,12	17,18	7,9.3	X,Y	6,11	9,12
1.00(36/36)	CRL-9482	BBM	12,13	13,13	10,13	12,12	17,18	7,9.3	X,Y	6,11	9,12
1.00(36/36)	CRL-9483	BZR	12,13	13,13	10,13	12,12	17,18	7,9.3	X,Y	6,11	9,12
1.00(36/36)	CRL-9609	BEAS-2B	12,13	13,13	10,13	12,12	17,18	7,9.3	X,Y	6,11	9,12
0.67(24/36)	CRL-7065	Hs 97.Fs	11,12	12,13	10,13	12,12	16,16	7,9.3	X,Y	8,11	10,12
0.61(22/36)	CRL-11233	THLE-3	13,13	13,13	8,10	11,12	17,18	8,9.3	X,X	6,9	11,12

BEP2D cells information was not found in DSMZ and ATCC.

Note: Reference standards: ANSI/ATCC, Authentication of Human Cell Line Standardization of STR Profiling. 2011, ASN - 0002-0002. STR results matches more than 80% can be considered the same source of cells.

3.3 BEP2D-Lqw STR Profiles

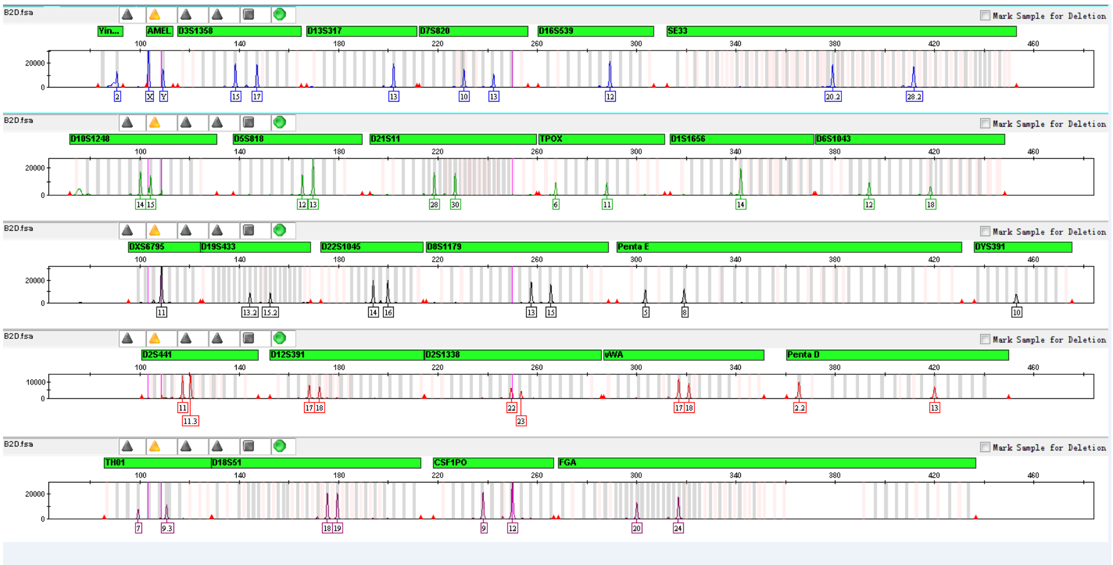

4. Reference

[1] Zhao, M., et al., Assembly and initial characterization of a panel of 85 genomically validated cell lines from diverse head and neck tumor sites. Clin Cancer Res, 2011. 17(23): p. 7248-64.

[2] Masters, J.R., Cell-line authentication: End the scandal of false cell lines. Nature, 2012. 492(7428): p. 186.

[3]American Type Culture Collection Standards Development Organization Workgroup, A.S.N., Authentication of Human Cell Lines: Standardization of STR Profiling. 2011, ANSI/ATCC ASN-0002-2011.

[4] Reid, Y.A., Characterization and authentication of cancer cell lines: an overview. Methods Mol

Biol, 2011. 731: p. 35-43.
[5] Lorsch, J.R., F.S. Collins, and J. Lippincott-Schwartz, Cell Biology. Fixing problems with cell lines. Science, 2014. 346(6216): p. 1452-3.
[6] Chatterjee, R., Cell biology. Cases of mistaken identity. Science, 2007. 315(5814): p. 928-31.
